# Assessing non‐adjunctive CGM safety at home and in new markets (ANSHIN)

**DOI:** 10.1002/edm2.414

**Published:** 2023-03-02

**Authors:** Christy Chao, Sarah B. Andrade, Simon Bergford, Peter Calhoun, John B. Welsh, Tomas C. Walker, ANSHIN Study Group

**Affiliations:** ^1^ Dexcom, Inc. San Diego California USA; ^2^ Jaeb Center for Health Research Tampa Florida USA

**Keywords:** blood glucose self‐monitoring, glycated haemoglobin, glycaemic control

## Abstract

**Introduction:**

Continuous glucose monitoring (CGM) can guide treatment for people with type 1 (T1D) and type 2 diabetes (T2D). The ANSHIN study assessed the impact of non‐adjunctive CGM use in adults with diabetes using intensive insulin therapy (IIT).

**Materials and Methods:**

This single‐arm, prospective, interventional study enrolled adults with T1D or T2D who had not used CGM in the prior 6 months. Participants wore blinded CGMs (Dexcom G6) during a 20‐day run‐in phase, with treatment based on fingerstick glucose values, followed by a 16‐week intervention phase and then a randomized 12‐week extension phase with treatment based on CGM values. The primary outcome was change in HbA1c. Secondary outcomes were CGM metrics. Safety endpoints were the number of severe hypoglycaemic (SH) and diabetic ketoacidosis (DKA) events.

**Results:**

Of the 77 adults enrolled, 63 completed the study. Those enrolled had mean (SD) baseline HbA1c of 9.8% (1.9%), 36% had T1D, and 44% were ≥65 years old. Mean HbA1c decreased by 1.3, 1.0 and 1.0 percentage points for participants with T1D, T2D or age ≥65, respectively (*p* < .001 for each). CGM‐based metrics including time in range also improved significantly. SH events decreased from the run‐in period (67.3 per 100 person‐years) to the intervention period (17.0 per 100 person‐years). Three DKA events unrelated to CGM use occurred during the total intervention period.

**Conclusions:**

Non‐adjunctive use of the Dexcom G6 CGM system improved glycaemic control and was safe for adults using IIT.

## Graphical abstract

ANSHIN is a single‐arm, prospective, interventional study that assessed the impact of non‐adjunctive Dexcom G6 continuous glucose monitoring (CGM) in adults with diabetes using intensive insulin therapy. After initiating CGM use, severe hypoglycemic events decreased and mean HbA1c decreased by 1.3, 1.0, and 1.0 percentage points for participants with type 1 diabetes (T1D), type 2 diabetes (T2D), or age ≥65, respectively (*p*<.001 for each) after 16 weeks. Non‐adjunctive use of CGM improved glycemic control and may help those with T1D or T2D make safe treatment decisions.

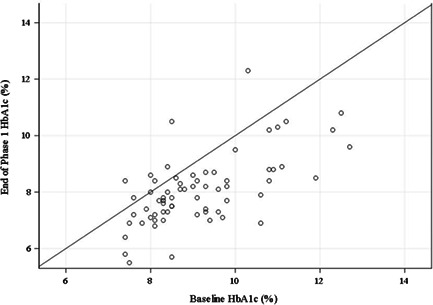

## 1 INTRODUCTION

Prior to the introduction of continuous glucose monitoring (CGM) systems, patients with type 1 diabetes (T1D) and type 2 diabetes (T2D) relied on self‐monitored blood glucose (SMBG) data to make therapy adjustments. While studies showed that SMBG improved glycaemic control, glucose management was still limited by the frequency of fingerstick measurements.^1^, ^2^ Real‐time CGM addressed this limitation by automatically measuring and transmitting glucose measurements to a receiver or mobile device every 5 min, thus providing full‐time glucose monitoring along with glucose trending information.^2^ CGM offers several additional advantages over SMBG, including programmable alerts and alarms, remote data sharing, and access to retrospective data.^3^, ^4^ These features have been shown to help people with diabetes safely manage their glucose levels.^5^, ^6^

Early CGM systems such as the Dexcom G4 were approved for adjunctive use, meaning that confirmatory SMBG measurements were still required to make insulin dosing decisions. Advancements to CGM accuracy and performance led to an expanded indication for the Dexcom G5, and in 2016, the US Food and Drug Administration (FDA) approved G5 data for non‐adjunctive use. The 2017 REPLACE‐BG study further demonstrated that using CGM data in place of SMBG testing as the basis for diabetes treatment decisions was safe for adults with T1D.^7^ In addition, the COACH^8^ study and the DIAMOND^5^ and MOBILE^9^ randomized clinical trials demonstrated that CGM use improved glycaemic control in people with diabetes better than SMBG, including greater reduced HbA1c, fewer SH events and improved CGM‐based metrics.

The Dexcom G6 system is approved for non‐adjunctive use in several jurisdictions for patients ages 2 years and older with insulin‐treated T1D or T2D. Previous studies have evaluated the sensor's accuracy,^10^, ^11^ and G6 data are now widely used to control automated insulin delivery systems.^12^, ^13^ Since receiving FDA clearance in 2018, the G6 signal processing algorithm was updated to increase data availability.^14^ The updated algorithm decreased the mean duration of data gaps by 47% and total time in sensor error by 59% while preserving the accuracy of the predicate algorithm.

Here, we report results from the Assessing Non‐adjunctive CGM Safety at Home and In New Markets (ANSHIN) study that evaluated the impact of non‐adjunctive use of this CGM system on glycaemic control in adults with intensive insulin therapy (IIT)‐managed diabetes. We assessed the change in HbA1c and CGM‐measured outcomes in participants who switched from SMBG testing to non‐adjunctive CGM use. The main safety endpoints were severe hypoglycaemia (SH) and diabetic ketoacidosis (DKA).

## 2 MATERIALS AND METHODS

The ANSHIN study was a prospective, interventional study with a single‐arm primary phase (phase one) and a randomized secondary phase (phase two). Participants underwent two sequential 10‐day run‐in periods using blinded CGM (G6 Pro, Dexcom, Inc.) to collect baseline data. During these run‐in periods, participants used SMBG data (Ascensia Contour NEXT 1 m) for diabetes treatment decisions. After run‐in, participants entered phase one of the study, a 16‐week intervention period during which participants were instructed to use CGM non‐adjunctively unless the CGM values did not align with symptoms or expectations. Participants were provided with a locked Android smartphone equipped with the Dexcom G6 commercial application, the Share/Follow application to share CGM data with others, and the Dexcom Clarity application to view and interact with CGM data. Initial high and low alerts were set to 13.9 mmoL/L and 3.9 mmoL/L, respectively; alerts for signal loss and upcoming hypoglycaemia were enabled. Participants could use the applications and features at their discretion. Study staff did not provide education about the use of CGM data nor were participants instructed to change their insulin treatment regimens. The study staff also did not provide guidance to participants or their healthcare providers to titrate toward specific glycaemic targets. Participants who completed the first phase were invited to continue to phase two.

**FIGURE 1:**
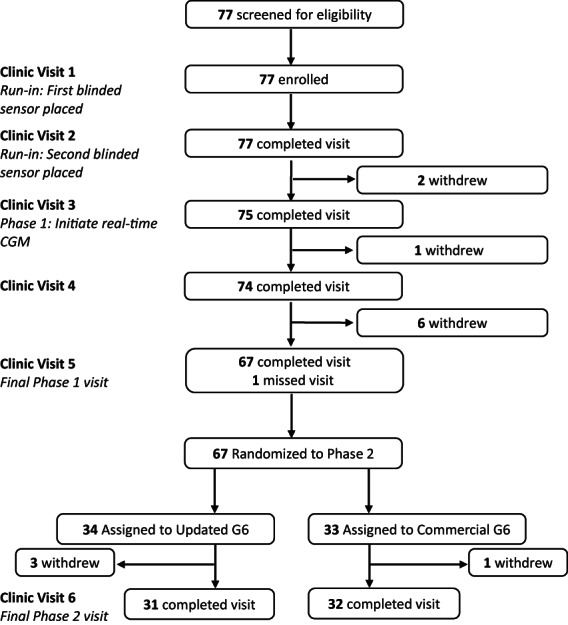
Flowchart of clinic visits and study completion.

In phase two, participants were randomly assigned (1:1) to wear two systems with either an updated^14^ or a commercially‐approved algorithm, with systems placed on both the posterior arm and abdomen, for 12 weeks. One of the CGM systems was blinded. A flowchart of the clinic visits is shown in Figure 1. The protocol and consent forms were approved by an Institutional Review Board (Advarra), and written informed consent was obtained from each participant. The study was conducted in accordance with the Declaration of Helsinki and was registered at clinicaltrials.gov (NCT04585139).

Adults aged 18 and older with T1D or T2D were enrolled at five sites in the United States. Participants were eligible if they had been diagnosed at least six months prior to enrollment, had used IIT to manage their diabetes for the last ≥3 months, had an HbA1c value of ≥7.5% within 30 days prior to enrollment or at screening, were currently performing ≥2 SMBG measurements per day, were willing to use the study‐provided SMBG meter and were willing to participate in both phases of the trial. Exclusion criteria included use of a CGM in the six months prior to enrollment (with the exception of professional blinded CGM), BMI >45 kg/m^2^, pregnancy, renal insufficiency (evidenced by eGFR <30 mL/min/1.73 m^2^), anticipated changes to insulin delivery method or insulin formulations, use of weight reduction medications or procedures, use of glucocorticoids, and medical conditions that could interfere with study‐related tasks (at the investigator's discretion).

The primary outcome was the change in HbA1c from the baseline SMBG monitoring period to the end of phase one. Secondary outcomes for both phases were CGM metrics (e.g., time in range [TIR], time below range [TBR] and time above range [TAR]). CGM metrics calculated at baseline included all blinded CGM data during the 20‐day run‐in period and CGM metrics calculated at phase one follow‐up included all unblinded CGM data in the 20 days prior to 16‐week follow‐up visit. CGM metrics were computed if there were at least 10 days of CGM data in the respective period. In addition to calculating overall results, the impact of CGM intervention was examined for the following subgroups: T1D, T2D and older adults (≥65 years).

The changes in study endpoints from baseline to the end of phase one were compared using two‐sided paired *t*‐tests. Wilcoxon signed‐rank tests were used to analyse skewed data. The primary outcome tested change in HbA1c for all participants with a significance level of 0.05. For testing changes in HbA1c within subgroups and testing secondary outcomes, the false discovery rate was controlled using the adaptive two‐stage Benjamini–Hochberg procedure.^15^ Safety analysis included the frequency of severe DKA events requiring treatment at a healthcare facility and SH events requiring the assistance of another person to resolve. All enrolled participants were included in the safety analysis.

The study was 90% powered to detect a difference in mean HbA1c between baseline and the end of phase one, assuming a mean difference of 0.4% from baseline, a common standard deviation of 0.7%, a correlation of 0.4, a significance level of 0.05 and using a two‐sided paired *t*‐test. To ensure sufficient power for primary analysis and subgroup analysis among older adults, an overall enrollment goal was set of 70 adults and 32 adults ≥65 years old. The sample size for phase two consisted of participants that completed phase one and continued into phase two.

## 3 RESULTS

A total of 77 participants were enrolled in the ANSHIN study, and 67 participants continued through phase two (Table 1). In phase one, there were 28 (36%) participants with T1D and 49 (64%) with T2D. In phase two, there were 25 (37%) with T1D and 42 (63%) with T2D. Mean (SD) HbA1c at enrollment was 9.8% (1.9%), and 44% of participants were ≥65 years old (45% in phase two). CGM utilization was high overall with 96% median percent time using G6 in phase one and 94% in both treatment groups in phase two. CGM data were missing and excluded from analysis for six participants that initiated G6 use but did not have any CGM record. Two participants withdrew from the study during the phase one run‐in period, seven withdrew during the phase one intervention period, and four withdrew during the phase two intervention period (Figure 1).

**TABLE 1:** Participant characteristics.

Demographic	Phase 1 (*N* = 77)	Phase 2 (*N* = 67)
Age, years (%)		
18‐ < 21	8 (10%)	6 (9%)
21‐ < 35	6 (8%)	6 (9%)
35‐ < 65	29 (38%)	25 (37%)
≥65	34 (44%)	30 (45%)
Mean ± SD	57 ± 19	57 ± 19
Range	18–88	18–88
Diabetes Type, *N* (%)		
Type 1	28 (36%)	25 (37%)
Type 2	49 (64%)	42 (63%)
Diabetes Duration, years (%)^a^		
<5	2 (3%)	2 (3%)
5‐ < 10	7 (9%)	5 (7%)
≥10	50 (65%)	45 (67%)
Mean ± SD	21 ± 12	22 ± 13
Range	5–59	5–59
Insulin Modality, *N* (%)		
Overall		
Insulin Pump	4 (5%)	4 (6%)
MDI	73 (95%)	63 (94%)
Type 1		
Insulin Pump	4 (14%)	4 (16%)
MDI	24 (86%)	21 (84%)
Type 2		
Insulin Pump	0 (0%)	0 (0%)
MDI	49 (100%)	42 (100%)
BMI, kg/m^2^		
Mean ± SD	30 ± 7	30 ± 7
Range	19–44	19–44
Sex—Female, *N* (%)	40 (52%)	35 (52%)
Race/Ethnicity, *N* (%)		
White non‐Hispanic	41 (53%)	34 (51%)
Hispanic or Latino	7 (9%)	6 (9%)
Black/African American	5 (6%)	4 (6%)
Asian	18 (23%)	17 (25%)
More than one race	6 (8%)	6 (9%)
HbA1c, *N* (%)		
7.5%‐ < 8.0%	7 (9%)	7 (10%)
8.0%‐ < 9.0%	23 (30%)	23 (34%)
≥9.0%	47 (61%)	37 (55%)
Mean ± SD	9.8 ± 1.9	9.5 ± 1.5
Range	7.5–16.1	7.5–14.4
≥1 DKA in Last 6 Months, *N* (%)	7 (9%)	NA
≥1 SH in Last 6 Months, *N* (%)	3 (4%)	NA

^a^ 18 Participants in phase one and 15 participants in phase two had missing diabetes duration.

**FIGURE 2:**
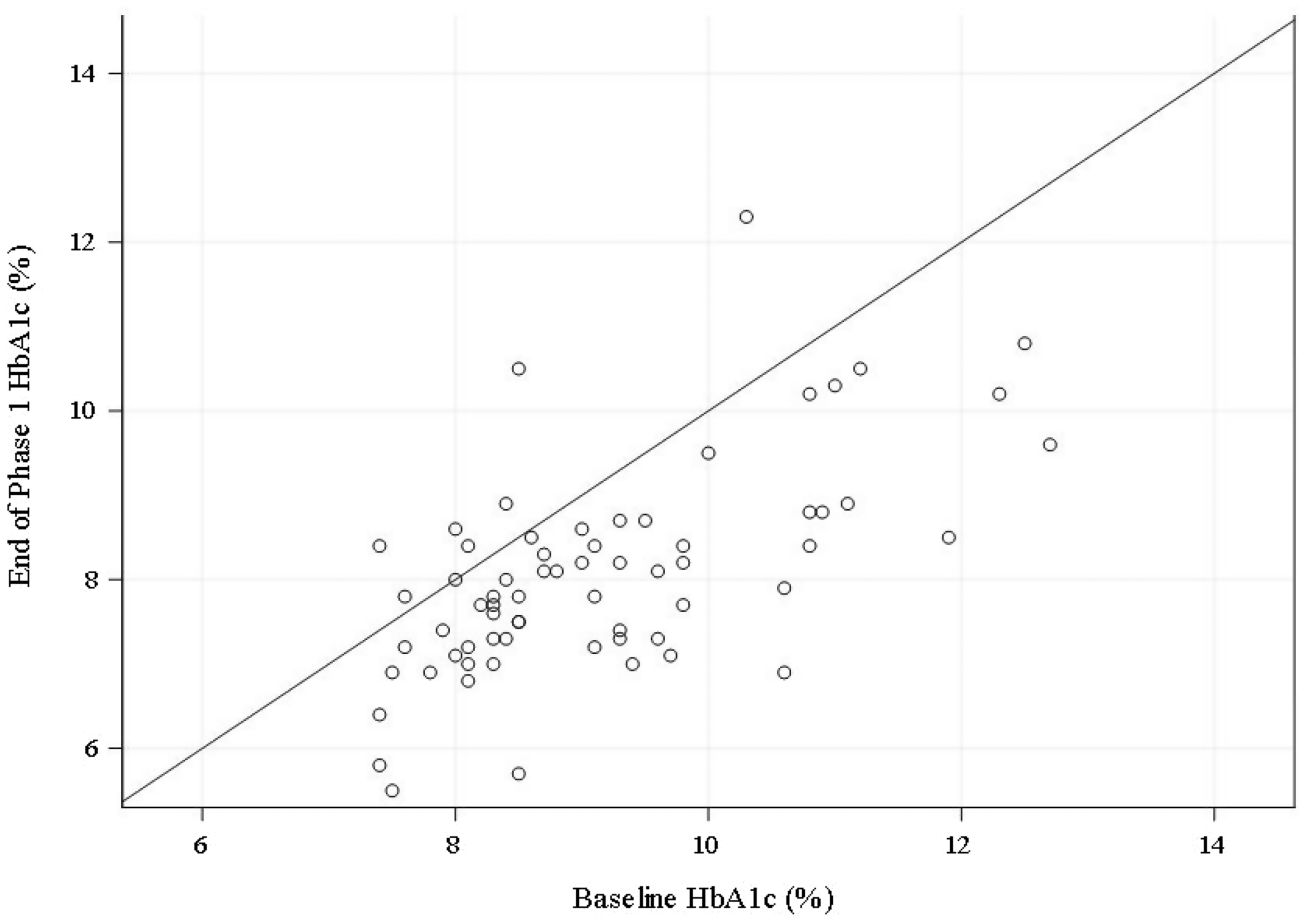
Scatter plot for HbA1c (%) at end of phase one versus baseline.

Mean HbA1c was reduced by 1.1 percentage points (*p* < .001), from 9.4% (1.5%) at baseline to 8.1% (1.2%) at the end of phase one (Table 2). Figure 2 shows the change in HbA1c levels during the phase one intervention period. Participants in both the T1D or T2D cohorts achieved significantly lower HbA1c levels from baseline to the end of phase one. For participants with T1D, mean HbA1c level decreased by 1.3 percentage points (*p* < .001) to 7.8% (1.2%) with non‐adjunctive CGM use. For participants with T2D, mean HbA1c level decreased to 8.2% (1.3%) with a mean reduction of 1.0 percentage points (*p* < .001) (Table 2). During the extension period, mean HbA1c reductions in participants with T1D or T2D seemed sustained but statistical significance was not determined (Table 4).

**TABLE 2:** Comparison of baseline and end of phase one glycaemic outcomes stratified by participants with T1D or T2D.

	Overall				T1D				T2D			
	Baseline	End of phase 1	Change	*p*‐Value	Baseline	End of phase 1	Change	*p*‐Value	Baseline	End of phase 1	Change	*p*‐Value
*N*	71	64	60	NA	25	23	20	NA	46	41	40	NA
HbA1c (%)	9.4 ± 1.5 (*N* = 74)	8.1 ± 1.2 (*N* = 67)	−1.1 ± 1.1 (*N* = 66)	<.001^a^	9.4 ± 1.7 (*N* = 27)	7.8 ± 1.2 (*N* = 25)	−1.3 ± 0.9 (*N* = 24)	<.001	9.3 ± 1.4 (*N* = 47)	8.2 ± 1.3 (*N* = 42)	−1.0 ± 1.2 (*N* = 42)	<0.001
CGM Outcomes												
Hours of CGM Data	422 ± 65	437 ± 53	17 ± 76	NA	417 ± 68	440 ± 50	22 ± 57	NA	425 ± 63	436 ± 55	15 ± 85	NA
Time in range 3.9–10.0 mmoL/L (%)	34 ± 18	47 ± 21	13 ± 18	<.001	33 ± 18	48 ± 22	15 ± 18	.004	34 ± 18	47 ± 21	12 ± 18	<.001
Mean glucose (mmoL/L)	12.5 ± 2.8	10.9 ± 2.4	−1.3 ± 1.9	<.001	12.7 ± 3.1	10.8 ± 2.5	−1.8 ± 2.3	.005	12.4 ± 2.7	11.1 ± 2.4	−1.1 ± 1.8	<.001
Glucose CV (%)	34 ± 9	33 ± 7	−1.5 ± 7.1	.11	38 ± 10	38 ± 8	0.0 ± 8.4	.98	32 ± 8	30 ± 5	−2.2 ± 6.3	.03
Hyperglycaemia												
Time >10.0 mmoL/L (%)	64 ± 19	51 ± 21	−13 ± 19	<.001	64 ± 20	49 ± 23	−15 ± 19	.005	65 ± 19	53 ± 21	−11 ± 19	<.001
Time >13.9 mmoL/L (%)	36 ± 23	23 ± 21	−11 ± 16	<.001	39 ± 23	25 ± 20	−13 ± 19	.010	34 ± 23	22 ± 21	−10 ± 14	<.001
Rate of hyperglycaemic events per week, >10.0 mmoL/L	13 ± 5	16 ± 5	2.5 ± 4.5	<.001	13 ± 5	15 ± 5	1.1 ± 5.1	.37	13 ± 5	16 ± 5	3.3 ± 4.1	<.001
Rate of hyperglycaemic events per week, >13.9 mmoL/L	12 ± 4	11 ± 6	−1.8 ± 5.4	.01	12 ± 4	12 ± 6	−1.2 ± 5.7	.34	12 ± 4	10 ± 6	−2.1 ± 5.3	.02
Hypoglycaemia, median (IQR)												
Time <3.9 mmoL/L (%)^b^	0.5 (0.0, 1.7)	0.7 (0.2, 1.8)	0.00 (−0.76, 0.60)	.76	0.6 (0.1, 5.4)	1.6 (0.6, 4.3)	0.01 (−2.14, 1.75)	1.00	0.3 (0.0, 1.2)	0.4 (0.0, 0.9)	0.00 (−0.56, 0.57)	.85
Time <3.0 mmoL/L (%)^b^	0.04 (0.00, 0.53)	0.07 (0.00, 0.50)	0.00 (−0.31, 0.07)	.17	0.17 (0.00, 1.92)	0.31 (0.04, 0.91)	0.00 (−0.99, 0.31)	.43	0.00 (0.00, 0.30)	0.05 (0.00, 0.19)	0.00 (−0.26, 0.06)	.24

*Note*: Data are mean ± SD or median (IQR). CGM outcome calculation requires ≥240 h of CGM data in the respective period. *p*‐values from a paired *t*‐test or Wilcoxon signed‐rank test, as appropriate, using available cases only. *p*‐values adjusted using the Benjamini–Hochberg procedure to control the false discovery rate.

^a^ *p*‐Value from a paired *t*‐test using available cases only.

^b^ *p*‐Values from Wilcoxon signed‐rank test.

Older participants ≥65 years also achieved significant HbA1c reductions after the first CGM intervention phase. Mean HbA1c for older adults decreased to 8.2% (1.4%) after non‐adjunctive CGM use with a mean reduction of 1.0 percentage points (*p* < .001; Table 3). At the end of the second phase, mean HbA1c level was 8.1% (1.1%) in those using the updated G6 algorithm (Table 4). Older participants that used commercial G6 showed some deterioration in mean HbA1c after phase two although statistical significance was not determined.

**TABLE 3:** Comparison of baseline and end of phase one glycaemic outcomes for participants ≥65 years.

	Baseline	End of phase 1	Change	*p*‐Value
*N*	32	30	28	NA
HbA1c (%)	9.3 ± 1.4 (*N* = 34)	8.2 ± 1.4 (*N* = 30)	−1.0 ± 1.2 (*N* = 30)	<.001
CGM Outcomes				
Hours of CGM Data	420 ± 56	435 ± 58	21 ± 78	NA
Time in Range 3.9–10.0 mmoL/L (%)	32 ± 17	46 ± 21	13 ± 20	.002
Mean Glucose (mmoL/L)	12.7 ± 3.0	11.2 ± 2.7	−1.3 ± 2.1	.003
Glucose CV (%)	34 ± 10	31 ± 6	−2.8 ± 7.3	.05
Hyperglycaemia				
Time >10.0 mmoL/L (%)	65 ± 19	53 ± 21	−12 ± 21	.005
Time >13.9 mmoL/L (%)	37 ± 24	23 ± 23	−12 ± 16	.001
Rate of hyperglycaemic events per week, >10.0 mmoL/L	12 ± 5	15 ± 5	2.7 ± 4.5	.005
Rate of hyperglycaemic events per week, >13.9 mmoL/L	11 ± 4	10 ± 5	−2.1 ± 5.3	.04
Hypoglycaemia, median (IQR)				
Time <3.9 mmoL/L^a^	0.2 (0.0, 1.7)	0.6 (0.1, 1.3)	0.00 (−1.03, 0.38)	.42
Time <3.0 mmoL/L (%)^a^	0.00 (0.00, 0.79)	0.06 (0.00, 0.23)	0.00 (−0.55, 0.03)	.09
Rate of hypoglycaemic events per week, <3.9 mmoL/L^a^	0.38 (0.00, 1.73)	0.82 (0.00, 2.01)	0.00 (−0.36, 0.41)	.96
Rate of hypoglycaemic events per week, <3.0 mmoL/L^a^	0.00 (0.00, 0.98)	0.00 (0.00, 0.36)	0.00 (−0.62, 0.00)	.03

*Note*: Data are mean ± SD or median (IQR). CGM outcome calculation requires ≥240 h of CGM data in the respective period. *p*‐values from a paired *t*‐test or Wilcoxon signed‐rank test, as appropriate, using available cases only. *p*‐values adjusted using the Benjamini–Hochberg procedure to control the false discovery rate.

^a^ *p*‐Values from Wilcoxon signed‐rank test.

**TABLE 4:** Glycaemic outcomes from end of phase one to end of phase two stratified by diabetes type and age.

	End of phase 1		End of phase 2		Change	
	Updated G6	Commercial G6	Updated G6	Commercial G6	Updated G6	Commercial G6
Overall						
HbA1c						
*N*	34	33	31	32	31	32
Mean ± SD (%)	8.1 ± 1.2	8.0 ± 1.3	8.3 ± 1.0	8.2 ± 1.5	0.30 ± 0.85	0.16 ± 0.75
Time in Range 3.9–10.0 mmoL/L						
*N*	33	31	30	31	29	29
Mean ± SD (%)	46 ± 21	48 ± 22	46 ± 17	51 ± 22	−1 ± 12	−1 ± 14
T1D						
HbA1c						
*N*	10	15	9	15	9	15
Mean ± SD (%)	7.8 ± 1.0	7.9 ± 1.3	7.9 ± 1.0	7.8 ± 1.6	0.19 ± 0.34	−0.07 ± 0.81
Time in Range 3.9–10.0 mmoL/L						
*N*	10	13	9	15	9	13
Mean ± SD (%)	45 ± 24	50 ± 21	48 ± 20	50 ± 22	−1 ± 6	−2 ± 10
T2D						
HbA1c						
*N*	24	18	22	17	22	17
Mean ± SD (%)	8.3 ± 1.3	8.1 ± 1.2	8.5 ± 1.0	8.5 ± 1.4	0.34 ± 0.99	0.35 ± 0.64
Time in Range 3.9–10.0 mmoL/L						
*N*	23	18	21	16	20	16
Mean ± SD (%)	47 ± 20	47 ± 23	45 ± 16	51 ± 22	−1 ± 14	0 ± 17
Age ≥65 years						
HbA1c						
*N*	17	13	15	12	15	12
Mean ± SD (%)	8.1 ± 1.6	8.5 ± 1.3	8.1 ± 1.1	8.9 ± 1.4	0.31 ± 0.81	0.27 ± 0.84
Time in Range 3.9–10.0 mmoL/L						
*N*	17	13	14	12	14	12
Mean ± SD (%)	51 ± 22	41 ± 18	50 ± 19	48 ± 23	−1 ± 13	5 ± 17

Between baseline and the end of the first phase, TIR improved significantly. CGM outcomes for those with T1D or T2D are summarized in Table 2 and outcomes for older adults are summarized in Table 3. Mean TIR for participants with T1D increased by 15 percentage points (*p* = 0.004; Table 2). Mean (SD) TIR for participants with T2D increased by 12 percentage points to 47% (21%) TIR at the end of phase one, similar to 48% (22%) TIR for those with T1D. Participants ≥65 years old experienced a 13‐percentage point TIR increase to 46% (21%) TIR by the end of phase one (*p* = .002; Table 3). There were similar significant reductions in TAR and mean glucose for participants with T1D or T2D and participants ≥65 years. There was no significant change in time below 3.9 or 3.0 mmoL/L for participants in any of these subgroups. The rate of hypoglycaemic events per week was very low but not significantly altered for participants with T1D or T2D (not shown).

There were three SH events from three participants during the 20‐day run‐in period, four SH events from four participants during the phase one CGM intervention period, and no SH events during phase two. SH events were unrelated to CGM use and were resolved by assisted intervention or, in one milder case, self‐treatment. The SH incidence rate was 67.3 events per 100 person‐years at baseline and 17.8 events per 100 person‐years during phase one, a 74% decrease. There were no DKA events during 20‐day run‐in period and two DKA events from one participant with T1D during phase one that were unrelated to CGM use. There was one additional DKA event from one participant with T2D during phase two that was unrelated to CGM use.

While this study did not aim to assess CGM accuracy, we report that the Dexcom G6 systems placed on the posterior arm tended to produce lower glucose values than systems placed on the abdomen. Arm‐derived glucose values were lower with median (IQR) difference of −0.3 (−1.1, +0.4) mmoL/L.

## 4 DISCUSSION

The ANSHIN study assessed the impact of non‐adjunctive Dexcom G6 use on glycaemic control and participant safety in people with suboptimal diabetes control (baseline mean HbA1c, 9.4%; mean TIR, 34%). In this study, significant improvements in HbA1c and CGM metrics and reduced SH events were observed following non‐adjunctive CGM use. Of note is that participants with T2D showed similar improvements in glycaemic outcomes as their counterparts with T1D. The results from the ANSHIN study will help inform regulatory agencies in Asian‐Pacific markets evaluating the safety and efficacy of this CGM system.

CGM is increasingly used by people with T2D to manage their glucose levels. Results from the ANSHIN study further support the benefit of CGM in this population. In this study, participants with T2D experienced a mean HbA1c reduction of 1.0 percentage points and a significant 12‐percentage point increase in TIR which corresponds to an increase of 2.9 h per day (*p* < .001). Several studies have evaluated the impact of the Dexcom G6 on glycaemic control. In the Landmark Study, adults with T1D or T2D who used IIT reported significantly decreased mean HbA1c from 8.2% to 7.1% alongside reduced diabetes distress and hypoglycaemic concerns in the first three months of initiating the G6.^16^ In the MOBILE study, adults with poorly‐controlled T2D using the G6 reported a 1.1‐percentage point reduction in mean HbA1c and increased TIR (59% versus 43% in SMBG group) after 8 months.^9^ Another study of patients with T2D treated with less intensive therapies observed a significant 3% reduction in mean HbA1c and 15‐percentage point increase in TIR in participants who used G6 for six months.^17^

Older adults are at greater risk of developing diabetes‐related complications,^18^ and analysis of the subgroup of participants who were ≥65 years old found that non‐adjunctive G6 use led to a 1.0‐percentage point reduction in HbA1c and a 3.2‐hour per day increase in TIR (mean increase of 13 percentage points; *p* = .002) alongside improvements in TAR and mean glucose. The results from this analysis are consistent with other publications assessing the impact of CGM in older participants. A sub‐analysis of the DIAMOND study reported that older adults with T1D or T2D using CGM achieved a significant 0.4% greater mean reduction in HbA1c and increased mean TIR (889 min versus 732 min in SMBG group) after 24 weeks.^19^ The WISDM study found that older participants with T1D using CGM over a six‐month period experienced a modest but significant reduction in time in hypoglycaemia (27 fewer minutes) compared to those in the blood glucose self‐monitoring group.^20^ A sub‐analysis of the MOBILE study found that older participants using CGM experienced a 19% greater increase in TIR and a 0.65% greater reduction in HbA1c compared to those using blood glucose meters.^21^

Participants in the ANSHIN study had fewer episodes of SH while using the G6 compared to the baseline SMBG period. This observation aligns with the COACH study that reported non‐adjunctive Dexcom G5 use was associated with reductions in hypoglycaemic events for patients with insulin‐treated T1D or T2D.^8^ Hypoglycaemia mitigation may be partly attributable to CGM alerts and alarms that warn users of impending low glucose. An analysis of real‐world G6 users demonstrated a reduction in hypoglycaemia following the introduction of the ‘Urgent Low Soon’ alert feature.^22^ Additionally, updated G6 algorithm use did not result in SH or DKA events, demonstrating that the updated algorithm was safe to use while also producing glycaemic outcomes similar to commercial algorithm users.

A strength of this analysis is the racial and ethnic diversity of the study population and the inclusion of 18 (23%) participants of Asian ancestry. The main limitation of the ANSHIN study is the lack of a non‐CGM control group during the intervention phases. Some of the reduction in HbA1c could be due to a study effect or regression to the mean phenomenon. However, there was a large mean (SD) HbA1c reduction of 1.1% (1.1%) achieved in a short period of time. Secondary CGM outcomes also provided additional evidence of a large improvement in glycaemia. A second limitation is that the study enrolled participants with suboptimal glycaemic control so findings are not generalizable to individuals with lower HbA1c levels. Third, while the total intervention duration was 28 weeks, this study only measured the change in glycaemic outcomes during phase one (16 weeks). Real‐world evidence suggests that glycaemic improvements are sustainable^23^ and additional clinical studies such as the ALERTT1 trial have assessed the long‐term benefits of CGM in maintaining lowered HbA1c and increased TIR.^24^

Finally, while participants experienced large glycaemic improvement, mean HbA1c remained above 8%, mean TIR was 47%, and mean TAR was 51% by end of study, falling short of the standard recommended glycaemic targets (HbA1c <7%, TIR >70%, TAR <25%).^25^ This may be partly due to the very poor glycaemic control exhibited at baseline compared to other studies in which baseline HbA1c was below 9%,^5^, ^26^ or even below 8%,^27^ and CGM metrics were closer to recommended guidelines at study onset.^26^, ^27^ However, this study's outcome is similar to the MOBILE study in which about a third of the CGM user group still exhibited a HbA1c level over 8% and had only 59% TIR after 8 months despite significant HbA1c reductions.^9^ The finding in the current study also broadly aligns with findings from a trial assessing glycaemic impact of intermittently scanned glucose monitoring. Participants in the FLASH‐UK trial significantly decreased HbA1c by 0.8 percentage points to 7.9% and increased TIR to 52% after 24 weeks but still did not meet recommended guidelines.^26^ Of note is that participants in the FLASH‐UK study received three sessions of diabetes management education while those in this current study received none. This study suggests that additional CGM education or clinical intervention may benefit those with ongoing suboptimal control.

Non‐adjunctive Dexcom G6 use improved glycaemic control in adults managing their diabetes with IIT, regardless of age and diabetes type. Significant improvements in HbA1c and CGM‐based outcomes were observed for participants with T1D, with T2D, and those ≥65 years of age. Non‐adjunctive CGM use was safe and was associated with a decrease in the rate of severe hypoglycaemic events. Initiating this CGM system may help those with T1D or T2D make safe treatment decisions and move toward improved glycaemic outcomes.

## AUTHOR CONTRIBUTIONS

**Christy Chao:** Writing – original draft (equal). **Sarah B. Andrade:** Writing – original draft (equal); writing – review and editing (equal). **Simon Bergford:** Formal analysis (equal). **Peter Calhoun:** Formal analysis (equal). **John B. Welsh:** Writing – original draft (equal); writing – review and editing (equal). **Tomas C. Walker:** Project administration (equal); supervision (equal); writing – review and editing (equal).

## AUTHOR DISCLOSURE STATEMENT

CC, SBA, JBW, and TCW are employees of Dexcom, Inc. PC's employer has received consulting payments on his behalf from vTv Therapeutics, Beta Bionics, Dexcom, and Diasome. SB has nothing to disclose.

## Data Availability

Authors elect to not share data.
